# Seeking Help From Trusted Adults in Response to Peers’ Social Media Posts About Mental Health Struggles: Qualitative Interview Study Among Latinx Adolescents

**DOI:** 10.2196/26176

**Published:** 2021-09-15

**Authors:** Celeste Campos-Castillo, Brian Jason Thomas, Felipe Reyes, Linnea Irina Laestadius

**Affiliations:** 1 Department of Sociology University of Wisconsin-Milwaukee Milwaukee, WI United States; 2 Zilber School of Public Health University of Wisconsin-Milwaukee Milwaukee, WI United States

**Keywords:** adolescents, confidants, ethnicity, gender, network resources, privacy, race, social media, social support, tie activation, trust

## Abstract

**Background:**

Rather than confiding in adults about their mental health struggles, adolescents may use social media to disclose them to peers. Disclosure recipients are tasked with deciding whether to alert an adult and, if so, whom to alert. Few studies have examined how adolescents decide on a trusted adult to help a friend who posts on social media about his/her mental health struggles. Moreover, Latinx adolescents are underrepresented in research on social media use, which creates gaps in understanding how social media may influence their well-being.

**Objective:**

This qualitative study presents findings from semistructured interviews with Latinx adolescents to investigate how they seek out trusted adults when a friend posts on social media about their mental health struggles. Specifically, we sought to determine which adult ties they activated, the resources they believed the adult could provide, and the support they expected the adult to provide.

**Methods:**

We recruited participants through a nonprofit organization serving the Latinx community (primarily of Mexican origin) located in Milwaukee, Wisconsin. We conducted 43 semistructured interviews, each lasting 60-90 minutes, with Latinx adolescents (25 females, 18 males) aged 13-17 years. All interviews were conducted in English, at the adolescents’ request. Using a grounded theory approach, we identified the nature of the relationship between the trusted adult and either the disclosure recipient or distressed friend, and the resources and support the trusted adult is expected to provide.

**Results:**

Participants nominated adults who were emotionally or physically proximate to either the disclosure recipient or distressed friend, particularly parents (of the recipient and friend) and school staff. However, some felt that not all parents and school staff were emotionally proximate. Adolescents sought trusted adults with access to two resources: experiential knowledge and authority. Some, particularly males, avoided adults with authority because of the risk of punishment and others thought their immigrant parents did not have relevant experiential knowledge to assist them. Interviewees felt that trusted adults with either resource could provide emotional and instrumental support either directly or indirectly, while those with experiential knowledge could provide informational support. Notably, interviews did not problematize the fact that the disclosure occurred on social media when deliberating about adults.

**Conclusions:**

To assist a distressed friend posting on social media, Latinx adolescents look not only for trusted adults who are emotionally and physically proximate but also those who have key resources that facilitate support. Efforts should focus on connecting adolescents with trusted adults and training adults who hold positions of authority or experiential knowledge to offer both direct and indirect support. Additionally, efforts should consider how immigrant experiences shape parent-child relations and address the potential long-term consequences of oversurveillance of Latinx youth, particularly males, by school staff for their access to social support.

## Introduction

Adolescents who disclose their struggles with depression or anxiety to peers on social media may be experiencing clinically significant symptoms [1]. Disclosure on social media affords access to social relationships for receiving social support and managing mental well-being [2-4]. Because some posts with mental health disclosures from adolescents may warrant adult notification and intervention [5], it is critical to understand the perspectives of the peers who receive these disclosures on social media. Adolescent disclosure recipients are charged with shielding the original poster from negative peer judgment (eg, bullying, gossip, and rumors) and potential punishment from adult authority figures (eg, parents and teachers) [6,7]. Disclosures on social media magnify the urgency of successful shielding because the medium facilitates rapid transmission of content in a way that sacrifices its original context and intended meaning [8-10]. Nonetheless, because adolescents may not seek help for mental health on their own [11-13], disclosure recipients are also faced with the burden of deciding whether concerning posts should be shared with an adult and, if so, with whom. In short, disclosure recipients must decide whether concealing from or disclosing to an adult would be better for protecting a friend who publishes concerning posts on social media.

Adults within the network of the disclosure recipient then likely serve as critical resources for responding to concerning posts. For example, adults may operate as gatekeepers who mediate access to mental health services [12]. The mere presence of adults, however, does not guarantee the provision of support because they may be deemed inappropriate for the health concern in question [14]. Some adults may exacerbate distress by questioning the credibility of the mental health concern [15,16], while others may have positive intentions to assist but be ill-equipped to respond because of limited guidance and training [17,18]. In addition, the occurrence of the disclosure on social media would further complicate the issue. Adults could punish either the disclosure recipient or distressed adolescent, perhaps for using social media outside of agreed upon rules or for any illegal, unethical, or risky behavior described in the post. Accordingly, disclosure recipients likely selectively activate ties with adults in their own network to marshal resources on behalf of friends who make mental health disclosures on social media. Concurrent with previous reports, we refer to such adults as “trusted adults” [19,20].

While prior studies have conceptualized trusted adults as having traits such as empathy, confidentiality, and physical and emotional availability, they focused on adolescents seeking help for themselves rather than for a friend [19-21]. One study on American Indian and Alaskan native youth examined how they would respond to concerning posts from a friend and offered initial evidence about the traits and roles of trusted adults [18]. A deeper understanding of how adolescents navigate their own networks to seek help for a friend who publishes concerning posts on social media is key for supporting the mental well-being of adolescents and may also reveal gaps in the resources that adolescents can marshal.

This study focused on how Latinx adolescents view trusted adults, specifically how they determine which adult ties to activate and the support they expect the adult to provide when a friend makes a hypothetical disclosure on social media about mental health struggles. Despite being one of the fastest growing demographic groups in the United States and segments of social media users [22,23], adolescents are underrepresented in research on social media use [8,24]. This potentially overlooks how social media can be beneficial for the mental wellness of adolescents. Latinx adolescents experience high rates of depression; nonetheless, they face socioeconomic and cultural barriers to receiving care [25]. Moreover, concerns over stigmatization shape who they seek for mental health support [26], which likely influences their perspectives on trusted adults. We therefore conducted qualitative interviews with Latinx adolescents to address gaps in studies on trusted adults and social media.

## Methods

The institutional review board at the University of Wisconsin-Milwaukee approved the study procedures. We collaborated with the United Community Center (UCC), a nonprofit organization serving the Latinx community in Milwaukee, Wisconsin, to recruit participants from a summer youth program. The Latinx population in Milwaukee is primarily of Mexican origin [27]. Like other Latinx youth in the United States, those in Milwaukee face disparities in mental health and access to trusted adults [27,28]. Prior to the start of the study, we conducted a focus group session with 15 Latinx youth at UCC to identify common mental health concerns (eg, feeling like a failure, suicidal thoughts, and unpleasant termination of relationships) to reference during the interviews. UCC screened eligible participants, who were Latinx adolescents aged 13-17 years who posted on social media at least once per week. We collected signed assent and consent forms from all adolescents and their guardians, respectively, before beginning with data collection. Assent and consent forms were available in both English and Spanish. Participants who enrolled in the study received an incentive of US $40.

We conducted 60-90–minute, semistructured, in-person interviews with 43 Latinx adolescents (25 females, 18 males) in a quiet room at UCC from June to July 2019. A Spanish-speaking interviewer was available, but all interviews were conducted in English at the request of the adolescents. The interview began with participants providing demographic information and selecting a pseudonym, which we use here to quote and summarize responses. After answering questions on social media and mental health disclosures, participants completed a card-sorting task presenting their mental health concerns. Informed by prior studies on how individuals make privacy decisions [29,30], participants were asked to separate the items that they would disclose to an adult, if they witnessed an adolescent friend express them on through social media posts, and to discuss general rules guiding their decisions. Participants were asked open-ended questions on their choice of the adult they would tell and why. This approach ensured that all participants were able to reflect on hypothetical situations that they personally felt warranted adult intervention. All interviews were digitally recorded and professionally transcribed verbatim. Interviewers wrote memos following each interview to reflect on findings, which the study team reviewed to identify emergent patterns and revise the interview guide accordingly.

A grounded theory approach was used to analyze transcripts [31]. During initial coding, 2 senior members of the research team read the first 12 interviews for instances in which interviewees reflected their choice of adults they would tell and why. This resulted in codes capturing the rationales for seeking adult intervention, the adults they would seek and why, and the traits of ideal and flawed trusted adults. Through discussion, initial codes were transformed into a codebook that described a smaller number of focused codes. We used MAXQDA 2018 (VERBI Software GmbH) to apply the codebook to all transcripts.

During the focused coding stage, 11 transcripts were coded by at least 2 team members, with each transcript discussed code-by-code. When code definitions shifted through discussion and the constant comparative method, we recoded previously reviewed transcripts as needed. We then examined focused codes in relation to each other and developed key categories, around which the results are organized.

## Results

### Results Overview

**Figure 1:**
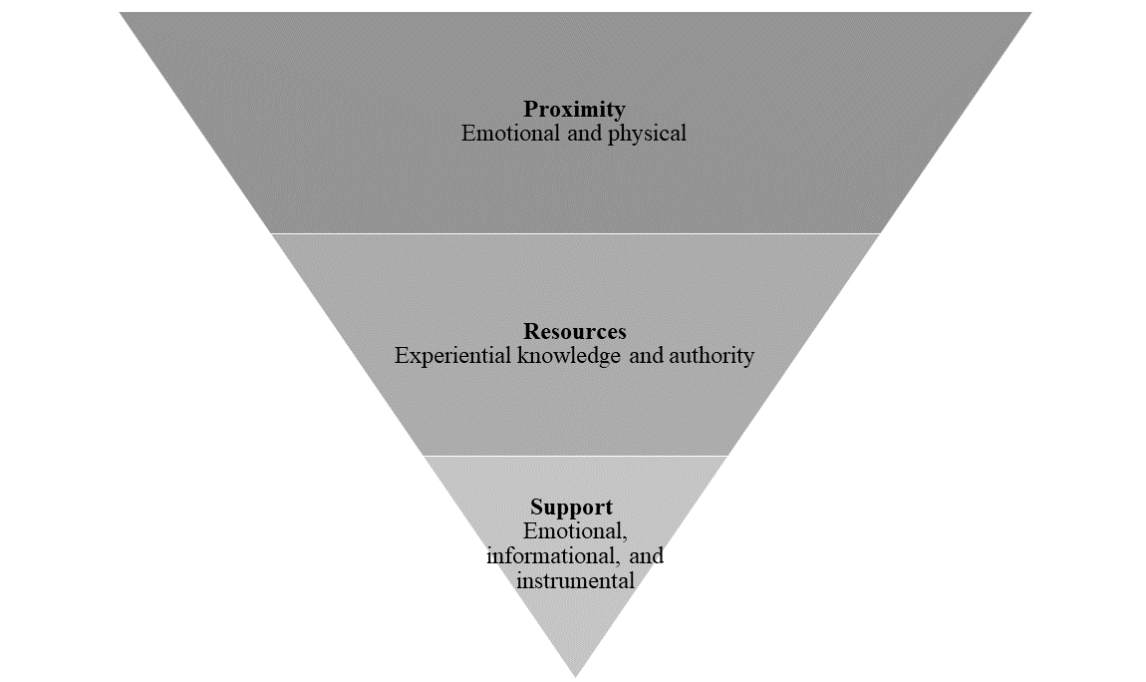
Categories that summarize how Latinx adolescents select a trusted adult to help with a friend who posted on social media about his/her mental health struggles.

Figure 1 shows the categories that summarize how Latinx adolescents select a trusted adult to provide support to a friend who posted about his/her mental health struggles on social media. The figure emphasizes how adolescents filter through possible adults to arrive at an ideal choice. We first describe the nature of the relationship (emotional and physical proximity) to either the disclosure recipient or the distressed friend and then present a model describing broad categories of resources interviewees sought out in trusted adults and the types of support they could provide. The overarching category of “resources” represents the resources that the interviewees seek when selecting a trusted adult: authority and experiential knowledge. The second category “support” captures the types of support the adolescents sought from trusted adults: emotional, instrumental, and informational.

### Nature of the Relationship

The most common types of trusted adults mentioned are indicated in Table 1, stratified by gender, which was the primary axis of difference. Across interviews, parents—of either the interviewee or the friend publishing a concerning social media post—were nominated. The interviewee’s own parents, particularly the mother, were commonly named. Alternatives to parents comprised adults at schools, including teachers, counselors, and principals. Overall, no individual type of adult was uniformly endorsed as optimal for assisting a friend posting on social media about being distressed. Instead, the nature of the relationship to either the disclosure recipient or distressed friend was key and varied across adult types.

**Table 1:** Frequencies and percentages of the type of adult nominated, stratified by the self-reported gender of the interviewee (N=43).

Type of adult	Females (n=25), n (%)	Males (n=18), n (%)	Total (n=43), n (%)
Own parent	22 (88)	12 (67)	34 (79)
Friend’s parent	14 (56)	10 (56)	24 (56)
Adult sibling	3 (12)	2 (11)	5 (12)
Other family member	3 (12)	4 (22)	7 (16)
Teacher	13 (52)	6 (33)	19 (44)
Counselor	10 (40)	5 (28)	15 (35)
Principal	5 (20)	2 (11)	7 (16)

#### Proximity to the Distressed Friend

Adolescents sought trusted adults with emotional and physical proximity to the distressed friend*.* This generally favored the parent of the friend over other adults, particularly because of an assumption that the parent was unaware of the affected adolescent’s mental health struggle. One participant noted, “I feel like I would really trust their parents because I feel like they themselves should know how their child is feeling” [Participant #41, female]. Some expressed practical concerns, which the physical proximity of the friend’s parent addressed. For example, while adolescents can proffer their own help, adolescents recognized limitations:

We’re not going to be there in person to watch them. So, if it’s a parent, they’re with them in the house, they make sure that that child is okay.

Participant #21, female

Some also felt that teachers inherently had a strong emotional proximity to students. One participant, for example, named teachers as a suitable choice of adult “since some of the teachers, well not some, all of the teachers care about you” [Participant #7, female].

However, emotional proximity of parents to their children was not always assumed. For example, a participant echoed others in believing that parents should know about a post, but then qualified his statement by stating that parents cannot be globally entrusted:

I feel like a parent…should know… I feel like it really depends. Because there might be some kids with a rough home life.

Participant #3, male

Others described proximity as having disadvantages. One participant reflected on the family dynamics within Latinx families and stated, “Especially a Latino, something about our parents just terrifies us” [Participant #20, male]. Further, the proximity of teachers and school staff to peers raised concerns among adolescents that reaching out to them would spur gossip and rumors.

#### Proximity to Disclosure Recipient

Participants also considered the physical and emotional proximity of the adult to themselves. Some participants, including one particular participant, felt that the parents of their friends were approachable:

I will most likely talk to their parents, especially since I know many of the parents of my friends. It would be really easy to reach out to them.

Participant #32, male

More commonly, however, participants identified their own parents as trusted adults because of proximity, with physical proximity breeding emotional proximity over time. One participant stated that she would tell her parents about a friend in need because of the following reason:

I really trust them. They’ve been taking care of me since I was little and they’ve always been there for me.

Participant #36, female

However, as with the parents of friends, several participants expressed concerns about their own parents, indicating physical proximity was insufficient for entrusting them.

### Resources

The interviews cited “authority,” which is the expectation that others would comply with the adult’s directives, as a resource of trusted adults. They saw authority as helpful in stopping a distressed friend from having harmful thoughts and behaviors, as determined from a participant’s interview: “[adults] can’t always stop suicidal thoughts; [they] can always stop suicide” [Participant #42, male]. Another participant wanted to activate ties with trusted adults with authority to immobilize those who were harming the distressed friend: “If they’re bullying you and you tell the teachers or your parents, the bullying is going to stop” [Participant #27, male].

Activating ties with a trusted adult with authority also had disadvantages. One participant, for example, was cautious about trusted adults using their authority to punish the disclosure recipient or distressed friend: “Because you never know like if they could just kick you out of their school for doing something like that” [Participant #4, female]. Others felt that adolescents such as themselves were overburdened by their required contact with school officials and were alienated because of the frequent exercise of authority over them, as one participant noted: “Because the school has done enough with us. Sometimes we just want a break from school in general” [Participant #20, male].

Adults in formal positions of authority offered an additional resource that adolescents were poised to activate: formal training. Several cited a distressed friend as falling under the authority of those who were formally trained in this domain by making comments such as it is “their job to help people.” One participant had a similar opinion and further stated that their formal training can determine the severity of the situation:

I would tell somebody at my school, like a teacher or a guidance counselor, about like, oh, this person is feeling this way. Like I don’t know if it's something super serious, but I feel like I see it that way. So I don’t know how you guys are going to handle this, but this is how somebody’s feeling.

Participant #18, female

Unlike expertise derived from training needed to assume a formal position of authority, experiential knowledge is gleaned from direct life experiences. Because the disclosure recipient is younger than an adult, interviews suggested that trusted adults “might know more than you do about how to react to these types of situations” [Participant #5, male]. Even those who occasionally seemed reticent to involve adults, owing to the assumption that they may not understand, conceded; for example, one participant stated that “they were teenagers at some point, so they know” [Participant #39, female]. In particular, disclosure recipients sought those with experiential knowledge germane to the problem that their friend was facing.

A signal influencing the perceived relevance of the experience was the similarity between the trusted adult and either the disclosure recipient or the distressed friend. This included having “been through something similar” or having a similar background, such as being proximate in age to the adolescents. One participant, for example, described why she would contact her adult sister rather than her parents:

[My parents] are not old but we also lived in different times. You know, like they would always mention to me like, ‘Oh, like in Mexico like they did this in school’…But…my older sister…we’re close so we-- I talk to her about a lot of things. She’s gone through similar things as I did, more similar than, you know, with what my parents have gone through.

Participant #19, female

The interview depicts another concern among Latinx adolescents with immigrant parents, which is that their parents’ experiences were irrelevant because of contextual differences in upbringing.

### Support

The interviews detailed different types of specific support the interviewees thought trusted adults could provide, which appeared related to the resources possessed. The first, emotional support, included listening and providing comfort and sympathy to the distressed friend. While not assumed to be present in every potential trusted adult, participants that felt both resources could yield emotional support. For example, one participant viewed an emotionally proximate adult, his adult sister, as possessing relevant experiential knowledge because they have “just grown up with each other” [Participant #23, male]. Consequently, he felt she could offer emotional support because they “literally tell each other everything” and that she provided “a safe environment” for communication. Adults with authority were also mentioned as providing emotional support. Another participant believed that by telling teachers, they could also be “on the lookout” within their classroom. Because they are physically proximate, teachers could observe a friend’s state. A teacher’s authority over a classroom grants them the ability to do more than observe and “lookout,” which would be helpful because one participant stated “they could sympathize” with his distressed friend [Participant #22, male].

Instances where interviews described tangible assistance were considered “instrumental support.” This was often related to formal positions of authority, where the trusted adult could curb harmful thoughts and behaviors. For trusted adults without a formal position, this included operating as a gatekeeper to contact others who did (eg, alerting teachers about cyberbullying perpetrated by a classmate) or to access formal avenues of support, including mental health services. Several participants stated that their most proximal contact, their parents, could reach out to the friend’s parents on their behalf. One participant, for example, stated, “I would tell my parents, and then they would probably talk to their parents about it or seek out help” [Participant #11, male].

The last type of support indicated here was “informational support,” which included guidance or advice and appeared to be related to trusted adults with experiential knowledge. In particular, trusted adults who endured circumstances similar to those that the friend encountered could offer input on how they overcame the ordeal. A participant interview explained that a distressed friend “could hear stories from somebody else who went through drugs who could have possibly died or were close to dying but survived and became someone greater than they were” [Participant #39, female]. One participant echoed the importance of hearing from someone who overcame one’s own struggle: “So they probably had mistakes that they have been through, so that they can tell them what to do next” [Participant #27, male].

## Discussion

### Principal Findings

We conducted interviews with Latinx adolescents to understand how they decide whom among their trusted adults they would engage with when concerned about a friend disclosing his/her mental health struggles on social media. Among the adults within their network, they considered the nature of the relationship between the adult and either themselves or their distressed friend and the resources and support the adult could provide. The findings clarify the resources and expected support sought by adolescents, while revealing circumstances potentially unique to Latinx adolescents. In turn, this study identifies prospective ways to foster adolescent-adult relationships and support access to resources mediated by adults.

The types of adults commonly nominated were parents, particularly the mothers of the disclosure recipient. Parents generally fit the stated criteria for trusted adults, which were being emotionally and physically proximate to either the disclosure recipient or distressed friend. This emphasis on proximity broadly echoes previous reports on the traits of trusted adults [21]. The focus on mothers complements other reports on the importance of mothers as confidants and for shaping mental health outcomes [32-34] by suggesting that they also serve as an important resource for their children’s friends. While not nominated as frequently, fathers are also important, especially in Latinx families where they tend to play a strong role in shaping screen time [35]. In instances where a parent was not nominated, this was due in part to parents immigrating to the United States and growing up in a context different from that of the adolescents. These findings suggest that culturally sensitive interventions aimed at addressing resource deficits should be tailored to the gender and immigration status of parents.

For adolescents concerned about contacting parents on behalf of a friend, adults at schools present an alternative. Teachers are charged with monitoring and assisting with the well-being of their students, such as confronting bullying between classmates, but students do not always depend on them [36]. Despite teachers’ physical proximity to both participants and distressed friends and the belief that their authority could halt harmful thoughts and behaviors, we found that some adolescents were reticent to alert teachers owing to a lack of emotional proximity. The reasoning they offered indicated feeling overburdened by the frequency of teachers exercising authority over them. For Latinx adolescents, particularly males, this may be a consequence of the harsh punishment and oversurveillance they endure compared to their White counterparts [37-40]. This presents both a gap in resources and a need to recognize and disrupt the potential long-term consequences of systemic racism within schools with respect to the social resources that adolescents can marshal when they or their peers are in need.

Our findings indicate that adolescents further vet trusted adults by the resources and support they can offer. Authority and experiential knowledge both represent areas where adolescents are traditionally lacking owing to both age and social dynamics. These 2 resources allow adolescents to mobilize adults to provide desired forms of support. However, one resource that was noticeably absent was digital literacy. Further, the interviews did not problematize the fact that the friend’s disclosure occurred on social media as they described their preferred adults. In this case it appears that, despite the social media context of the friend in distress, the key preconditions for activating an adult tie are whether the adult can offer emotional, instrumental, or informational support.

Studies such as ours on adolescents’ understandings of trusted adults can help inform interventions aimed at creating adolescent-adult connections and fostering resources and support skills among adults [19]. Adolescents deemed each type of adult nominated as capable of providing emotional support, suggesting that any trusted adult can be trained to provide it. While adolescents did not perceive every type of adult as providing instrumental support to assist a distressed friend, they did see the potential for someone to provide such support indirectly by mediating access to another adult who could. This suggests opportunities to provide guidance to adults about key contacts for mental health support. We subsequently intend to survey parents or guardians (ie, the most commonly nominated trusted adult) on their views of adolescents expressing mental health struggles on social media, perceived efficacy for supplying support, and awareness of resources to assist them, while focusing on identifying gaps that future interventions could address.

### Limitations

The limitations of this study include sampling of English-speaking Latinx adolescents from a summer program in a mid-sized US city whose residents are largely of Mexican origin. Such sampling potentially misses important differences by country of origin and language barriers faced in contacting adults outside of home. Focus on a setting in which Latinx adolescents report fewer adult confidants than other ethnoracial groups helped identify local gaps in resources. Another limitation is that the interviews largely described who would be contacted in hypothetical scenarios, thus limiting the ability to determine what events occur in an actual emergency. Our findings should be interpreted as describing normative ideals and in which case it is still illuminating to observe instances when certain types of adults were avoided.

### Conclusions

Adults within the network of adolescents may not always be well-informed about their well-being [32,34,36]. Instead, adolescents may use social media to confide in their peers, making it critical to understand how disclosure recipients may marshal adult support. Semistructured interviews with Latinx adolescents revealed how resources and support provided by trusted adults are linked. Not every adult physically proximate to an adolescent is considered trustworthy, and Latinx adolescents may experience gaps in access to trusted adults, thus reflecting their families’ immigrant experiences and long-term consequences of systemic racism. Future efforts should support forging connections between adolescents and adults and guide adults on ways in which they can provide different types of support.

## Abbreviations

UCC: United Community Center
